# Comparison of the effectiveness of clomiphene citrate, tamoxifen and letrozole in ovulation induction in infertility due to isolated unovulation

**Published:** 2012-11

**Authors:** Fariba Seyedoshohadaei, Farnaz Zandvakily, Shole Shahgeibi

**Affiliations:** *Department of Obstetrics and Gynecology, Besat Hospital, Kurdistan University of Medical Sciences, Sanandaj, Iran.*

**Keywords:** *Infertility*, *Unovulation*, *Non-polycystic ovarian syndrome*, *Ovulation induction*, *Clomiphene*, *Tamoxifene*, *Letrozole*

## Abstract

**Background:** Unovulation is the most common cause of infertility. The first line oral treatment has been clomiphene citrate. Another anti-estrogen used for ovulation induction is tamoxifen. Many unovulatory infertile women are resistance to anti-estrogens and need another treatment. Alternative treatments are aromatas inhibitors.

**Objective: **This study was designed to compare the effectiveness of clomiphene, tamoxifen and letrozole in ovulation induction outcomes in isolated non PCOS unovulatory patients.

**Materials and Methods:** 150 unovulatory infertile women who had isolated non- polycystic ovarian syndrome (PCOS), randomized to 3 groups. Group A received clomiphene 50 mg to maximum 150 mg for 5 days, Group B received tamoxifen 10mg to maximum 30 mg for 5 days, Group C received letrozole 2.5 mg for 5 days, to maximum 7.5 mg until ovulation was induced. If ovulation failed to occur with 5 days treatments, drug continued for 7 days. Treatment has been stopped if they became pregnant or if patient didn’t ovulate with maximum dose for 7 days (resistant to treatment) or failed to concept after six months despite ovulation (failure of treatment). Main outcome measures were: number of mature follicles, endometrial thickness, pregnancy rate, multiple pregnancy rate, live birth and miscarriage.

**Results:** Overall ovulation rate was 60 (73.4%), this rate in group A was 39 (78%), in group B it was 24 (68%) and in group C was 37 (74%). Pregnancy rate in groups A, B and C were, 32 (64%), 20 (40%), and 25 (50%) respectively, and live birth rate was 22 (44%) in A, 17 (34%) in B and 21 (42%) in C. Miscarriage rate with clomiphene was 10 (20%) while this was 3 (6%) in tamoxifen and 4 (8%) in letrozole group (p=0.05). One twin pregnancy was occurred with clomiphene and one with tamoxifen, while all pregnancies with letrozole were singleton.

**Conclusion:** Because of higher pregnancy rate with clomiphene citrate than tamoxifen and letrozole, Clomiphene citrate is still the first-line therapy for ovulation induction. Surprisingly, pregnancies after tamoxifen and letrozole have lower miscarriage rate than clomiphene.

## Introduction

The most common cause of infertility in women is anovulation. There are many drugs used for induction ovulation among isolated unovulation non-polycystic ovarian syndrome (PCOS). The first line oral treatment is non-steroidal selective estrogen receptor modulators (SERM) (1). Clomiphene citrate has been introduced in 1956 (2). That is the first-line method of ovulation induction in women with anovulatory infertility. Since 1962 it has been the drug of choice for oral ovulation induction over the last 50 years (3-5). Clomiphene can induce ovulation in 80% of anovulatory women but only 40% of women became pregnant (6). Pregnancy rate per cycle can be 10-20% (2) and as high as 60% after six cycles and 97% after 10 cycles (7). Unfortunately, 20-25% of the women are resistant to CC and fail to ovulate (8).

Another anti-estrogen used for ovulation induction is tamoxifen; there are no appreciable differences in ovulation or pregnancy rates after treatment with tamoxifen or clomiphene for isolated anovulatory infertility (6). Many unovulatory infertile women are resistance to anti-estrogens and need another treatment. Alternative treatments are aromatas inhibitors. “Aromatas is a cytochrome P-450 hemoprotein and catalyzes the rate-limiting step in the production of estrogens” (9). Letrozole, a highly selective AI, recently used for induction ovulation as alternative to CC in unovulatory infertile patients. (3-10)

Letrozole increases FSH levels and therefore increases in the number of multiple mature follicles (11, 10) and do not have adverse endometrial effects because that half life is shorter then clomiphene, so increased pregnancy rate (2). In most studies, letrozole used for induction ovulation in polycystic ovarian disease (PCOS), but data in non PCOS patients is limited.

The aim of this study is to compare the effectiveness of clomiphene, tamoxifen and letrozole for ovulation induction, endometrial thickness, and pregnancy rate, multiple pregnancy, live birth or miscarriage in isolated “non PCOD” unovulatory patient. 

## Materials and methods

This study was performed as a randomized single-blinded (researchers blinded) prospective controlled clinical trial in private clinics, Sanandaj, Iran. Between November 2007 and September 2009, a series of 150 women that admitted to private clinics included in the present study.

**Figure 1:**
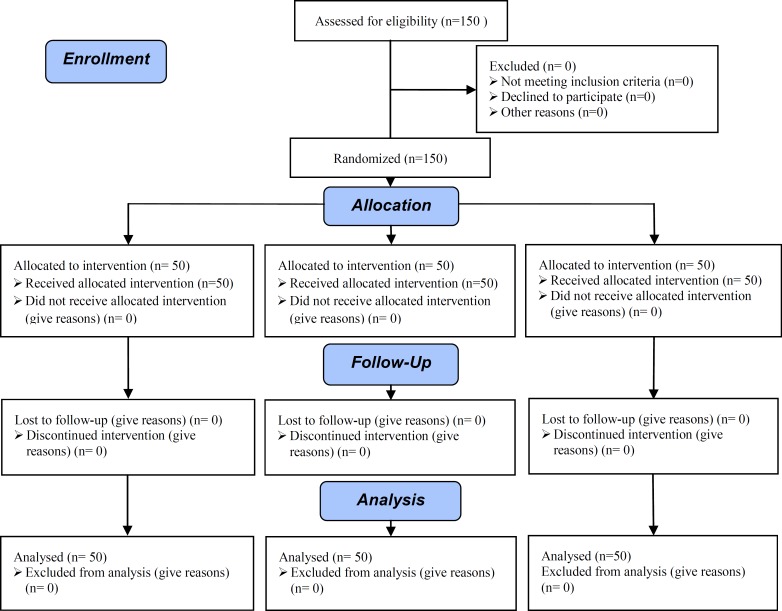
Consort flow diagram.

The inclusion criteria were patients with infertility of at least 1 year, menstrual cycle between 35 days to 6 months, normal serum prolactin, thyroid-stimulating hormone levels, follicular stimulating hormone, normal luteinizing hormone, normal uterus and ovary without evidence of polycystic ovary or dominant follicle at midcycle in ultrasonography, normal uterous and patent tubes on the hysterosalpingogram, and normal semen analysis for their husbands. Study participants were counseled, and informed consent was obtained from 150 non-PCOS unovulatory infertile women (isolated unovulation). They randomized to 3 groups by random table as Group A, B and C. (Figure 1. Consort flow diagram)

Group A (n=50) received initial clomiphene (Clomid, Iran Hormone Company) 50mg daily from day 3 of the menstrual cycle for 5 days. Group B (n=50) received initial tamoxifen 10mg daily (Tamoxifen, Iran Hormone Company), starting from day 3 of the menstrual cycle for 5 days. Group C received initial letrozole (Letrax, Aboryhan Farmacy) 2.5mg daily from day 3 of the menstrual cycle for 5 days after spontaneous or progesterone-induced menses. 

If ovulation failed to occur with the initial dose of either drug, the daily dosage was increased in group A by 50mg increment in subsequent cycle to 100 and 150 mg, in group B by 10mg increment in subsequent cycle to 20 and 30mg, and in group C by 2.5mg increment in subsequent cycle to 5 and 7.5mg in subsequent cycles. 

If ovulation failed to occur with 5 days treatments, drug continued for 7days. Treatment has been stopped if they became pregnant or if patient didn’t ovulate with maximum dose for 7 days (resistant to treatment) or failed to concept after six months despite ovulation (failure of treatment) and if they had intolerable symptoms (vision changes, depression, debilitating headaches, abdominal pain and hot flashes).

Ultrasonographyic examinations were performed on day 14 of the cycle to monitor the number and size of developing follicles and endometrial thickness. Main outcome measures were number of follicles ≥18mm, endometrial thickness and ovulation rate. Secondary outcome measures clinical pregnancy rates (serum βHCG over 10IU after missed period), spontaneous abortions rates (pregnancy loss before 20 weeks according to first trimester sonography), multiple pregnancies, and ovarian hyperstimulation syndrome (OHSS) (enlargement ovary with multiple cyst and acitis according to sonography). If they had retarded manses, serum βHCG was performed for diagnosis of pregnancy.

**Statistical analysis**

SPSS version 11(SPSS Inc., Chicago, IL, USA) was used X^2^ test was used to all parameters. Proportional variables were compared using the Fisher’s exact test. Normally distributed continuous variables were compared with the Student’s *t*-test. p*≤*0.05 was considered statistically significant.

## Results

Demographic characteristics in three groups of patient shows main age and duration of infertility were similar (Table I). A total of 567 cycles were studied in 150 patients. All of 150 participants completed the study and were included in the final analysis of data. (Table II). The cumulative pregnancy rate in three groups was 77 (51.3%) at 6 months. In compare with tamoxifen and letrozole pregnancy rate was higher with clomiphene. Although number of pregnancies were higher in the letrezole group than tamoxifen group, this difference was not significant. There were 17 (22%) pregnancies that ended in miscarriage and 83 (78%) of pregnant women successfully delivered. The clomiphene group have significantly higher miscarriage rate (Table III). 

Endometrial thickness on 14 cycles was higher with tamoxifen but not statistically significant (8.03±3.13 mm in group B vs. 7.7±4.15mm in group A and 6.07±2.76mm in group C). One twin pregnancy was occurred with clomiphene and tamoxifen, but all pregnancies with letrozole was singletons. No higher order pregnancies (triplet or higher pregnancy) and no ovarian hyperstimulation syndrome (OHSS) occurred with oral induction ovulation.

**Table I:** Patients’ characteristics in all three randomized groups (mean±SD)

	**Clomiphene citrate** **(group A)**	**Tamoxifen** **(group B)**	**Letrozole** **(group C)**	**p-value**
No. of patients	50	50	50	
Age (years)	24.72 (±4.66)	25.44 (±4.18)	26.94 (±4.59)	0.395
Duration of infertility	2.95 (±2.06)	2.99 (±2.03)	4.06 (±2.65)	0.906

*Note: *No significant differences between treatment t-test.

**Table II:** Results of treatment in isolated anovulatory women

** Month**		**1**	**2**	**3**	**4**	**5**	**6**	**Total cycles**
**Group**								
Group A (Clomiphene)								
	Number of patients	50	48	37	28	21	15	199 cycle
	Discontinuation*	-	4	3	1	-	-	8
	Pregnant patients	2	7	6	6	6	5	32
	Miscarriage	-	2	3	2	3	-	10
	Live birth	2	5	3	4	3	5	22
Group B (Tamoxifen)								
	Number of patients	50	48	31	16	15	13	174 Cycle
	Discontinuation	-	8	9	1	1	-	19
	Pregnant patients	2	9	6	-	1	2	20
	Miscarriage	-	3	-	-	-	-	3
	Live birth	2	6	6	-	1	2	17
Group C (Letrozole)								
	Number of patients	50	50	39	26	17	12	194 cycle
	Discontinuation	-	5	6	1	1	-	13
	Pregnant patients	-	6	7	8	4	-	25
	Miscarriage	-	1	2	1	-	-	4
	Live birth	-	5	5	8	4	-	22

* Discontinuation of treatment due to resistance to drugs (No ovulation with maximum dose). X^2 ^test.

**Table III:** Characteristics of outcome parameters of women treated three protocols

**Parameters**	**Group A (Clomiphene citrate)**	**Group B (Tamoxifen)**	**Group C (Letrozole)**
Ovulation rate	39 (78%)	34 (68%)	37 (74%)
Pregnancy rate	32 (64%)*	20 (40%)	25 (50%)
Live birth rate	22 (44%)	17 (34%)	21 (42%)
Miscarriage rate	10 (20%)	3 (6%)	4 (8%)

[Odds ratio (OR) 0.755, 95% confidence interval (CI) 0.513-1.111].

(p=0.05, X2=9.37)

* p=0.05 for clomiphene compared with letrozole and tamoxifen.

## Discussion

The present studies demonstrated that despite good result with letrozole in PCOS patients, that reported in many articles, clomiphene citrate is superior to letrozole and tamoxifen for induction ovulation in non-PCOD unovulatory women. Current prospective study illustrated a cumulative chance of pregnancy by classical ovulation induction with clomiphene in normogonadotrophic unovulatory infertile women is 71% within 2 years (12). In this trial cumulative pregnancy rate was 64% in non-PCOD unovulatory patients.

Abu Hashim said that “Clomiphene citrate is not equally effective in all situations for induction of ovulation” (13). Stephanie and coworkers demonstrated that the aromatase inhibitor “letrozole is equivalent to clomiphene for stimulation of follicular growth in normal ovulatory women” (2). Despite tamoxifen, significantly fewer follicles were observed in cycles stimulated with 2.5 mg letrozole compared with cycles stimulated with 100 mg clomiphene (14). Elnashar *et al* (2006) reported an ovulation rate of 54.6% and pregnancy rate of 25% with letrozole induction in clomiphene citrate -resistant women with PCOS (15). 

Ashalatha Ganesh *et al* (2009) reported the ovulation rate of 79.3% and the pregnancy rate of 23.39% with letrozole (4). This data is similar to ovulation rate of 37 (74%), and pregnancy rate of 29 (58%) in our study. Some investigators found that, no statistically significant difference between ovulation rates and pregnancy rates in tamoxifen compare to clomiphene (6-16). In this study pregnancy rate was higher with letrozole then tamoxifen although there were no significant differences between two groups. 

In our study, ovulation rate was same in three groups, but pregnancy rate was significantly higher with clomiphene then tamoxifen and letrozole. (p=0.05 X_2_=9.37) Clomiphene citrate is an anti-estrogen and has peripheral effect especially on endometrial thickness, while letrozole; because the short half-life; does not have peripheral anti-estrogen effect (2, 11). In our study, Letrozole cycles are associated with fewer total follicles and fewer mature follicles but more endometrial thickness compared with clomiphene citrate cycles. Abu Hashim *et al* reported that, endometrial thickness on 14 cycles had a no significant increase in the letrozole group (13). Surprisingly; Badawy *et al* (2007); reported significantly greater endometrial thickness in the CC group than letrozole (11). Endometrial thickness is higher, but not significant, with tamoxifen then clomiphene and letrozole. 

The rate of pregnancy loss after ovarian stimulation, with different protocols, was not higher than after spontaneous pregnancy. Many reports have referred to increased overall rates of miscarriage in infertile patients (17). We observed higher miscarriage rates in pregnancies after Clomiphene compared with pregnancy after tamoxifen and letrozole. Ruiz-Velasco *et al* reported a higher spontaneous abortion rate in their cohort of tamoxifen-treated patients compared with clomiphene-treated patients, whereas Boostanfar *et al* observed only one abortion in tamoxifen group and non in clomiphene group (16). But similar to our study, Wu Ch. reported lower miscarriage rate in pregnancy after tamoxifen as compared with clomiphene (18). 

Some investigator reported low multiple gestation rates after ovarian stimulation by aromatase inhibitors (19). In many patients especially in PCOS infertile women letrozole is ideal choice, because limited number of mature follicles, multiple pregnancies and risk of hyperstimulation syndrome (8, 20). Badawy *et al* reported that incidence of multiple pregnancies with oral induction ovulation is not significantly higher than normal ovulatory women (19). There are few reports of multiple births in the letrozole group (21). In our study, we observed two twin pregnancies, one in clomiphene group and one in tamoxifen group.

## Conclusion

Clomiphene citrate is still the first-line therapy for ovulation induction. Clomiphene, tamoxifen and letrozole show similarity in term of ovulation rate. There is no benefit of tamoxifen and letrozole over clomiphene citrate in pregnancy achievement. But patients were in higher risk of multiple births with clomiphene. Surprisingly miscarriage rate was lower if patient conceived with tamoxifen or letrozole than clomiphene.

## Footnote

***Registration ID in IRCT: IRCT201104096152N1***
